# Assessment of the quality of different commercial providers using artificial intelligence for automated cephalometric analysis compared to human orthodontic experts

**DOI:** 10.1007/s00056-023-00491-1

**Published:** 2023-08-29

**Authors:** Felix Kunz, Angelika Stellzig-Eisenhauer, Lisa Marie Widmaier, Florian Zeman, Julian Boldt

**Affiliations:** 1 Department of Orthodontics, University Hospital of Würzburg, Pleicherwall 2, 97070 Würzburg, Germany; 2 Centre for Clinical Studies, University Hospital of Regensburg, Regensburg, Germany; 3 Department of Prosthetic Dentistry, University Hospital of Würzburg, Würzburg, Germany

**Keywords:** Deep Learning, Machine Learning, Human gold standard, Cephalometric landmarks, Orthodontic parameters, Deep Learning, Machine Learning, Menschlicher Goldstandard, FRS Landmarken, Kieferorthopädische Parameter

## Abstract

**Purpose:**

The aim of this investigation was to evaluate the accuracy of various skeletal and dental cephalometric parameters as produced by different commercial providers that make use of artificial intelligence (AI)-assisted automated cephalometric analysis and to compare their quality to a gold standard established by orthodontic experts.

**Methods:**

Twelve experienced orthodontic examiners pinpointed 15 radiographic landmarks on a total of 50 cephalometric X‑rays. The landmarks were used to generate 9 parameters for orthodontic treatment planning. The “humans’ gold standard” was defined by calculating the median value of all 12 human assessments for each parameter, which in turn served as reference values for comparisons with results given by four different commercial providers of automated cephalometric analyses (DentaliQ.ortho [CellmatiQ GmbH, Hamburg, Germany], WebCeph [AssembleCircle Corp, Seongnam-si, Korea], AudaxCeph [Audax d.o.o., Ljubljana, Slovenia], CephX [Orca Dental AI, Herzliya, Israel]). Repeated measures analysis of variances (ANOVAs) were calculated and Bland–Altman plots were generated for comparisons.

**Results:**

The results of the repeated measures ANOVAs indicated significant differences between the commercial providers’ predictions and the humans’ gold standard for all nine investigated parameters. However, the pairwise comparisons also demonstrate that there were major differences among the four commercial providers. While there were no significant mean differences between the values of DentaliQ.ortho and the humans’ gold standard, the predictions of AudaxCeph showed significant deviations in seven out of nine parameters. Also, the Bland–Altman plots demonstrate that a reduced precision of AI predictions must be expected especially for values attributed to the inclination of the incisors.

**Conclusion:**

Fully automated cephalometric analyses are promising in terms of timesaving and avoidance of individual human errors. At present, however, they should only be used under supervision of experienced clinicians.

## Abstract (de)

**Ziel:**

Ziel dieser Untersuchung war es, die Analysequalität verschiedener kommerzieller Anbieter für KI(künstliche Intelligenz)-basierte Fernröntgenseitenanalysen (FRS-Analysen) zu untersuchen und deren Auswertungen mit einem durch Experten festgelegten Goldstandard zu vergleichen.

**Methoden:**

Auf 50 FRS wurden durch 12 erfahrene Untersucher 15 Landmarken identifiziert, auf deren Basis 9 relevante Parameter für die kieferorthopädische Behandlungsplanung vermessen wurden. Der „menschliche Goldstandard“ wurde definiert, indem der Medianwert aller 12 menschlichen Bewertungen für jeden Parameter berechnet wurde. Dieser diente als Referenzwert für den Vergleich mit den Ergebnissen von 4 verschiedenen kommerziellen Anbietern automatisierter FRS-Analysen (DentaliQ.ortho [CellmatiQ GmbH, Hamburg, Deutschland], WebCeph [AssembleCircle Corp, Seongnam-si, Korea], AudaxCeph [Audax d.o.o., Ljubljana, Slowenien], CephX [Orca Dental AI, Herzliya, Israel]). Die statistische Auswertung erfolgte mittels ANOVAs mit Messwiederholungen sowie mittels Bland-Altman-Plots.

**Ergebnisse:**

Die Ergebnisse der ANOVAs mit Messwiederholung zeigten signifikante Unterschiede zwischen den Vorhersagen der kommerziellen Anbieter und dem menschlichen Goldstandard für alle 9 untersuchten Parameter, wobei sich im Rahmen der anschließenden paarweisen Vergleiche große Unterschiede zwischen den 4 kommerziellen Anbietern ergaben. Während keine signifikanten Unterschiede zwischen den Werten von DentaliQ.ortho und dem Goldstandard festgestellt wurden, wichen die Vorhersagen von AudaxCeph bei 7 von 9 Parametern signifikant ab. Außerdem zeigten die Bland-Altman-Plots, dass grundsätzlich eine geringere Präzision der KI-Vorhersagen bei den Parametern für die Inklination der Frontzähne zu erwarten ist.

**Schlussfolgerung:**

Vollständig automatisierte FRS-Analysen sind vielversprechend in Bezug auf ihre Zeitersparnis und die Vermeidung individueller menschlicher Fehler. Derzeit sollten sie jedoch nur unter Aufsicht erfahrener Kliniker eingesetzt werden.

## Introduction

Artificial intelligence (AI) has become an integral part of our lives and, due to the increasing availability of computing power, can be used for increasingly complex tasks in medicine or dentistry [33]. In recent years, there has been an exponential increase in scientific publications aiming to integrate AI into daily orthodontic routine. These range from identification of anatomical or pathological structures and/or reference points in imaging all the way to support complex decision-making [16].

Particularly, AI algorithms have been successfully developed for automated evaluation of cephalometric images [1, 5, 7, 9, 12–14, 17–20, 25, 27, 28, 32, 33, 37]. Prior to the use of AI, specialized software that facilitated geometric constructions and measurements was available, but locating landmarks remained a manual task for the practitioner [24]. This time-consuming and error-prone process can be largely automated by AI. In 2021, Schwendicke et al. evaluated the accuracy of automated cephalometric landmark detection developed by different authors in a meta-analysis, demonstrating that the majority of the included models could identify landmarks within clinically acceptable tolerance [31].

Despite these promising approaches, Schwendicke et al. pointed out that there is elevated risk of bias in the majority of studies investigating the use of AI for the automated analysis of cephalometric images. This aspect gains importance in light of the fact that commercial providers already offer software solutions for automated analysis of cephalometric images without sufficiently disclosing the scientific basis of their AIs [31].

The aim of this investigation was therefore to evaluate the accuracy of various skeletal and dental cephalometric parameters as analyzed by currently available commercial providers for automated cephalometric analysis and to compare these assessments to a humans’ gold standard established by orthodontic experts.

## Patients and methods

The present investigation was carried out in compliance with the Declaration of Helsinki.

### Definition of a humans’ gold standard

All cephalometric X‑rays used for this study were recorded on a Sirona Orthophos XG (Dentsply Sirona, Bensheim, Germany) and were obtained from a private orthodontic practice. In order to comply with all applicable data protection regulations, all images were fully anonymized.

A total of 12 experienced orthodontic examiners (6 orthodontic specialists, 6 dentists in the second half of their post-graduate orthodontic education) at the Department of Orthodontics of the University Hospital of Würzburg pinpointed 15 radiographic landmarks on a total of 50 randomly selected (out of a pool of 3000) cephalometric X‑rays (Table 1). The images exhibited a great variety in terms of the dentition phase and orthodontic anomalies. Furthermore, some patients had fixed orthodontic appliances installed at the time of acquisition. The landmarks were used to generate nine commonly used parameters for orthodontic treatment planning (Table 2). On this basis, a “humans’ gold standard” for statistical comparisons was defined by calculating the median value of the 12 human assessments for each parameter. To determine intrarater reliability, 20 of the 50 cephalometric X‑rays were randomly selected and re-evaluated several weeks later by every examiner.

**Table 1:** Definitions of cephalometric landmarks Definitionen der kephalometrischen Referenzpunkte

Landmark	Abbreviation	Definition
Sella	S	Midpoint of the hypophyseal fossa
Nasion	N	Furthest anterior point of the nasofrontal suture in the median-sagittal plane
A‑point	A	Furthest posterior point of the curvature of the upper alveolar bone in the median-sagittal plane
B‑point	B	Furthest posterior point of the curvature of the lower alveolar bone in the median-sagittal plane
Anterior nasal spine	ANS	Furthest anterior point of the osseous anterior nasal spine in the median-sagittal plane (= furthest anterior point of the maxilla)
Posterior nasal spine	PNS	Radiological intersection of the anterior border of the pterygopalatine fossa and the skeletal base of the maxilla (= furthest posterior point of the maxilla)
Articulare	Ar	Radiological intersection of the lower margin of cranial base and dorsal margin of the mandibular ramus
P1-point	P1	Furthest caudal point of the curvature of the jaw angle of the mandible
P2-point	P2	Furthest posterior point of the curvature of the jaw angle of the mandible
Menton	Me	Furthest caudal point of the mandibular symphysis
Gonion	Go	Intersection of the Ar-P2 line and the Me-P1 line *(constructed landmark)*
Incision superior	I‑U1	Incisal edge of the upper central incisors (should more than one contour be visible, the anterior contour is defaulted to)
Incision inferior	I‑L1	Incisal edge of the lower central incisors (should more than one contour be visible, the anterior contour is defaulted to)
Apex superior	A‑U1	Furthest apical point of the upper central incisors
Apex inferior	A‑L1	Furthest apical point of the lower central incisors

**Table 2:** Definitions of cephalometric parameters Definitionen der kephalometrischen Parameter

Parameter	Unit	Definition and interpretation
*Skeletal sagittal analysis*		
SNA	°	Angle between sella, nasion and A‑point → Sagittal position of the maxilla
SNB	°	Angle between sella, nasion and B‑point → Sagittal position of the mandible
ANB	°	Angle between A‑point, nasion and B‑point → Skeletal class
*Skeletal vertical analysis*		
SN-PP	°	Angle between the anterior cranial base (= sella-nasion line) and the palatal plane (ANS-PNS line) → Inclination of the maxilla
SN-MeGo	°	Angle between the anterior cranial base (= sella-nasion line) and the mandibular plane (menton-gonion line) → Inclination of the mandible
PP-MeGo	°	Angle between the palatal plane (= ANS-PNS line) and the mandibular plane (menton-gonion line) → Skeletal open/deep bite
Facial height	%	Ratio between the posterior (= sella-gonion line) and anterior (= nasion-menton line) facial height → Skeletal growth pattern
*Dental analysis*		
U1-SN	°	Angle between the upper central incisors (= line between incision superior and apex superior) and the anterior cranial base (= sella-nasion line) → Inclination of the upper central incisors
L1-MeGo	°	Angle between the lower central incisors (line between incision inferior and apex inferior) and the mandibular plane (= menton-gonion line) → Inclination of the lower central incisors

The procedure to set the humans’ gold standard presented above has already been described in a previous investigation [17]. However, compared to the aforementioned study (in which a total of 12 parameters were examined using 18 radiological landmarks), three metric parameters were not included in this investigation as not all commercial providers supported these specific AI-based detections.

### Selection of the commercial providers for AI-assisted automatic cephalometric x-ray analyses

Selection of commercial providers for AI-assisted cephalometric X‑ray analysis was conducted in March 2021. The authors’ goal was to include all providers offering fully automated detection of cephalometric landmarks based on deep-learning algorithms. As an additional inclusion criterion, the landmarks defined in Table 1 and the parameters defined in Table 2 had to be included in the AI-assisted analyses of the providers.

Four commercial software providers were identified that met all inclusion criteria:

- DentaliQ.ortho (CellmatiQ GmbH, Hamburg, Germany),
- WebCeph (AssembleCircle Corp., Seongnam-si, Korea),
- AudaxCeph (Audax d.o.o., Ljubljana, Slovenia), and
- CephX (Orca Dental AI, Herzliya, Israel).

CephNinja (Cyncronus LLC, Bothell, WA, USA) was excluded as the requirements for landmarks and parameters were not met.

### Data acquisition

Data analysis was performed in March 2021 by the same experienced examiner (W., L. M.). The entire data set of 50 cephalometric X‑rays was imported into the software of all four providers and automatically analyzed. Subsequently, the resulting values for the nine cephalometric parameters were exported. This process was repeated in July 2021 to check whether unannounced updates to the AI may have resulted in altered assessments. No changes could be identified for any provider as exactly the same results were obtained in both evaluations. Three cephalometric X‑rays were retrospectively excluded from the statistical analyses due to interference caused by radiographic artefacts.

### Statistical analysis

Statistical analysis of the data was performed by a professional biometrician. All analyses were carried out using SPSS Statistics version 25.0 for Windows® (IBM, Ehningen, Germany) and R (version 4.1.0). For all statistical analyses, the level of significance was set to 5%.

Reliability of the analyses performed by the 12 experienced orthodontic examiners used to define the humans’ gold standard was verified using intraclass correlation coefficients (ICC). Interrater reliability was analyzed for each parameter and intrarater reliability was verified for each examiner and each parameter.

We calculated repeated measures analysis of variance (ANOVAs) to compare the analyses of the four commercial providers and the humans’ gold standard for each of the nine parameters. Post hoc pairwise comparisons between each provider and the humans’ gold standard were made using Bonferroni correction to control for alpha error.

In a second step, Bland–Altman plots were generated for all investigated parameters to illustrate differences between the predictions of each commercial provider and the humans’ gold standard (y-axis) versus the humans’ gold standard itself (x-axis) [15]. In these plots, the mean differences between both methods are used to assess the “average trueness” of the commercial providers. A mean difference to the humans’ gold standard of less than 0.5° was considered “good average trueness”, “moderate average trueness” if the mean difference was between 0.5 and 1.0°, and “low average trueness” if the mean difference exceeded 1.0° (or % for the parameter facial height). Furthermore, the 95% limits of agreement (= LoA/mean difference ± 1.96 × standard deviation of the differences) were used to evaluate the “precision” of the analyses of the commercial providers. In this regard, we considered the precision to be “good” if the standard deviation of the differences was less than 1.5°, “moderate” if it was between 1.5 and 2.5°, and “low” if it was more than 2.5° (or % for the parameter facial height). If both good average trueness and good precision were given, a method was deemed to have good accuracy. A linear regression line was added to each Bland–Altman plot to visually determine possible proportional biases.

## Results

### Reliability of the humans’ gold standard

Interrater reliability was very high for all parameters analyzed in this study (all ICC > 0.900 with *p* < 0.001). Likewise, intrarater reliability was very high for each examiner and each parameter (all ICC > 0.800 with *p* < 0.001).

### Results of the repeated measures ANOVAs and pairwise comparisons

The results of the repeated measures ANOVAs are depicted in Table 3. Pairwise comparisons between each provider and the humans’ gold standard (post hoc analysis) are depicted in Table 4. The results of the repeated measures ANOVAs indicate that there were significant differences between the four commercial providers and the humans’ gold standard for all nine investigated parameters. However, the pairwise comparisons demonstrate that there were also major differences concerning the average trueness of the analyses among the four commercial providers.

**Table 3:** Comparison of the predictions of the artificial intelligence (AI)-assisted commercial providers for cephalometric X‑ray analysis and the humans’ gold standard. Descriptive statistics with mean (M) and standard deviation (SD); repeated measures analysis of variance (ANOVA) with *p*-value (Greenhouse–Geisser) Vergleich der Auswertungen der kommerziellen Anbieter für KI(Künstliche Intelligenz)-basierte FRS-Analysen mit dem menschlichen Goldstandard. Deskriptive Statistik mit Mittelwert (M) und Standardabweichung (SD); Varianzanalyse mit Messwiederholung (ANOVA) und *p*-Wert (Greenhouse-Geisser)

Parameter	Unit	Group	M	SD	*p*
*Skeletal sagittal analysis*					
SNA	°	Humans’ gold standard	81.23	2.61	0.000**
		DentaliQ.ortho	81.12	2.54	
		WebCeph	81.29	2.81	
		AudaxCeph	82.59	2.70	
		CephX	81.58	2.58	
SNB	°	Humans’ gold standard	78.56	2.86	0.000**
		DentaliQ.ortho	78.60	3.02	
		WebCeph	78.36	3.09	
		AudaxCeph	79.67	2.87	
		CephX	78.64	2.94	
ANB	°	Humans’ gold standard	2.65	2.26	0.002**
		DentaliQ.ortho	2.52	2.07	
		WebCeph	2.93	1.82	
		AudaxCeph	2.94	2.10	
		CephX	2.95	2.10	
*Skeletal vertical analysis*					
SN-PP	°	Humans’ gold standard	7.33	2.78	0.000**
		DentaliQ.ortho	7.39	2.70	
		WebCeph	8.02	1.86	
		AudaxCeph	5.67	2.79	
		CephX	7.76	2.54	
SN-MeGo	°	Humans’ gold standard	30.62	7.07	0.000**
		DentaliQ.ortho	30.60	7.20	
		WebCeph	31.68	6.45	
		AudaxCeph	29.97	7.02	
		CephX	35.15	6.27	
PP-MeGo	°	Humans’ gold standard	23.38	6.34	0.000**
		DentaliQ.ortho	23.21	6.36	
		WebCeph	23.66	5.46	
		AudaxCeph	24.30	6.12	
		CephX	27.38	4.92	
Facial height	%	Humans’ gold standard	68.15	6.29	0.000**
		DentaliQ.ortho	68.21	6.35	
		WebCeph	66.94	5.43	
		AudaxCeph	69.65	6.10	
		CephX	63.08	4.94	
*Dental analysis*					
U1-SN	°	Humans’ gold standard	103.96	6.74	0.013*
		DentaliQ.ortho	103.87	6.04	
		WebCeph	103.04	4.80	
		AudaxCeph	103.64	5.08	
		CephX	104.96	5.89	
L1-MeGo	°	Humans’ gold standard	94.15	7.34	0.000**
		DentaliQ.ortho	93.82	6.55	
		WebCeph	93.81	4.65	
		AudaxCeph	94.65	6.04	
		CephX	88.19	5.52	

*Significance for *p* < 0.05

**Significance for *p* < 0.01

**Table 4:** Comparison of the predictions of artificial intelligence (AI)-assisted commercial providers for cephalometric X‑ray analysis versus the humans’ gold standard. Post hoc analysis of repeated measures analysis of variance (ANOVA) with mean difference, 95% confidence interval and *p*-value (*p*) Vergleich der Auswertungen der kommerziellen Anbieter für KI(Künstliche Intelligenz)-basierte FRS Analysen mit dem menschlichen Goldstandard. Post-hoc-Analyse der Varianzanalyse mit Messwiederholung (ANOVA) und *p*-Wert (*p*)

Parameter	Unit	Humans’ gold standard vs. commercial providers		Mean difference	95% Confidence interval		p
					Lower limit	Upper limit	
*Skeletal sagittal analysis*							
SNA	°	Humans’ gold standard vs	DentaliQ.ortho	−0.11	−0.56	0.33	1.000
			WebCeph	0.06	−0.91	1.02	1.000
			AudaxCeph	1.36	0.81	1.91	0.000**
			CephX	0.35	−0.19	0.89	0.602
SNB	°	Humans’ gold standard vs	DentaliQ.ortho	0.03	−0.38	0.44	1.000
			WebCeph	−0.21	−0.97	0.56	1.000
			AudaxCeph	1.10	0.62	1.59	0.000**
			CephX	0.08	−0.38	0.54	1.000
ANB	°	Humans’ gold standard vs	DentaliQ.ortho	−0.13	−0.42	0.17	1.000
			WebCeph	0.28	−0.20	0.76	0.874
			AudaxCeph	0.29	0.05	0.53	0.008**
			CephX	0.30	0.01	0.60	0.041*
*Skeletal vertical analysis*							
SN-PP	°	Humans’ gold standard vs	DentaliQ.ortho	0.06	−0.48	0.60	1.000
			WebCeph	0.69	−0.26	1.64	0.365
			AudaxCeph	−1.66	−2.28	−1.04	0.000**
			CephX	0.43	−0.40	1.27	1.000
SN-MeGo	°	Humans’ gold standard vs	DentaliQ.ortho	−0.02	−0.48	0.44	1.000
			WebCeph	1.06	−0.09	2.20	0.090
			AudaxCeph	−0.66	−1.29	−0.02	0.038*
			CephX	4.53	3.82	5.23	0.000**
PP-MeGo	°	Humans’ gold standard vs	DentaliQ.ortho	−0.16	−0.70	0.37	1.000
			WebCeph	0.28	−0.86	1.42	1.000
			AudaxCeph	0.92	0.45	1.39	0.000**
			CephX	4.01	3.17	4.85	0.000**
Facial height	%	Humans’ gold standard vs	DentaliQ.ortho	0.06	−0.33	0.45	1.000
			WebCeph	−1.20	−2.41	0.00	0.050
			AudaxCeph	1.50	0.83	2.17	0.000**
			CephX	−5.07	−5.89	−4.25	0.000**
*Dental analysis*							
U1-SN	°	Humans’ gold standard vs	DentaliQ.ortho	−0.09	−1.06	0.87	1.000
			WebCeph	−0.93	−2.96	1.11	1.000
			AudaxCeph	−0.32	−1.65	1.00	1.000
			CephX	1.00	−0.18	2.17	0.163
L1-MeGo	°	Humans’ gold standard vs	DentaliQ.ortho	−0.33	−1.35	0.70	1.000
			WebCeph	−0.33	−2.18	1.51	1.000
			AudaxCeph	0.51	−0.67	1.69	1.000
			CephX	−5.96	−7.45	−4.47	0.000**

*Significance for *p* < 0.05

**Significance for *p* < 0.01

#### Humans’ gold standard vs. DentaliQ.ortho

The results of DentaliQ.ortho were similar to the humans’ gold standard. No significant difference was found for any of the nine investigated parameters (all adjusted *p*-values = 1.000). The smallest absolute deviation from the humans’ gold standard was found for SN-MeGo (∆ = 0.02°) and the largest deviation was found for L1-MeGo (∆ = 0.33°).

#### Humans’ gold standard vs. WebCeph

Similar results were found for the values obtained from WebCeph with no significant differences for any of the nine investigated parameters. The largest absolute deviation (∆ = 1.20%) from the humans’ gold standard was found for facial height (*p* = 0.050). The highest average trueness was observed for the SNA-angle (∆ = 0.06°).

#### Humans’ gold standard vs. AudaxCeph

AudaxCeph’s analyses exhibited significant differences for all skeletal sagittal and skeletal vertical parameters (all adjusted *p*-values < 0.05). No significant differences were found for the dental parameters U1-SN and L1-MeGo (all adjusted *p*-values = 1.000). The smallest absolute deviation was found for the ANB-angle (∆ = 0.29°), whereas the largest was found for the parameter SN-PP (∆ = 1.66°).

#### Humans’ gold standard vs. CephX

In the pairwise comparisons, five out of the nine investigated parameters exhibited a significant difference to the humans’ gold standard. These were ANB (*p* = 0.041), SN-MeGo (*p* < 0.001), PP-MeGo (*p* < 0.001), facial height (*p* < 0.001), and L1-MeGo (*p* < 0.001). Moreover, the four latter parameters showed a rather large absolute deviation from the humans’ gold standard of more than 4° (or % for the parameter facial height), in case of L1-MeGo almost 6°. The smallest deviation was observed for the SNB-angle (∆ = 0.08°).

### Results of the Bland–Altman plots

The results of the Bland–Altman plots are depicted in Figs. 1, 2, 3, 4, 5, 6, 7, 8 and 9.

**Fig. 1:**
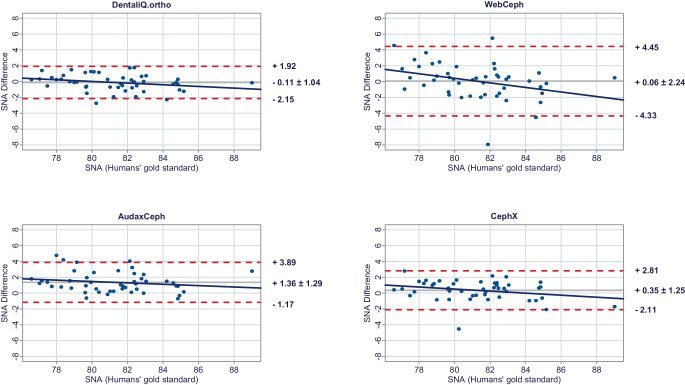
Bland–Altman plots comparing the analyses of the commercial providers to the humans’ gold standard: SNA Bland-Altman-Plots zum Vergleich der Analysen der kommerziellen Anbieter mit dem menschlichen Goldstandard: SNA

**Fig. 2:**
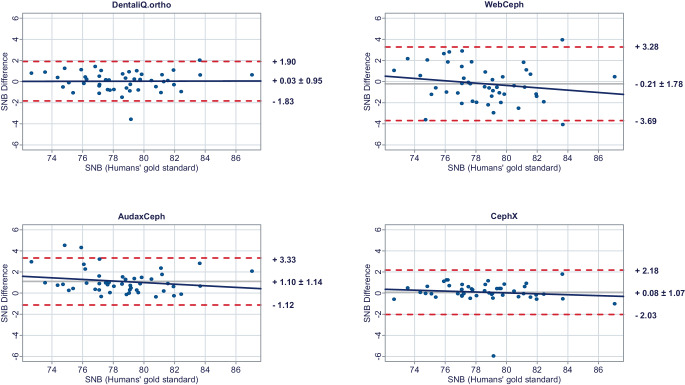
Bland–Altman plots comparing the analyses of the commercial providers to the humans’ gold standard: SNB Bland-Altman-Plots zum Vergleich der Analysen der kommerziellen Anbieter mit dem menschlichen Goldstandard: SNB

**Fig. 3:**
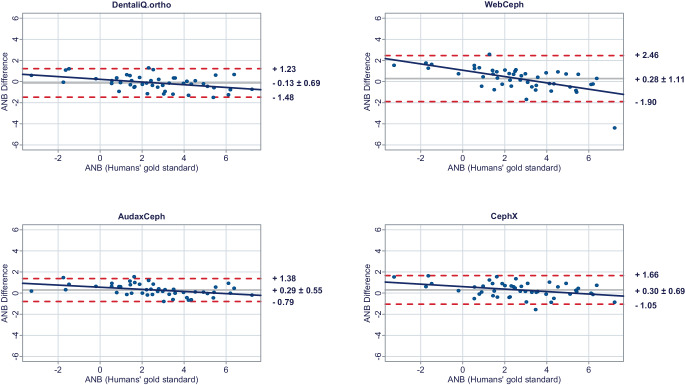
Bland–Altman plots comparing the analyses of the commercial providers to the humans’ gold standard: ANB Bland-Altman-Plots zum Vergleich der Analysen der kommerziellen Anbieter mit dem menschlichen Goldstandard: ANB

**Fig. 4:**
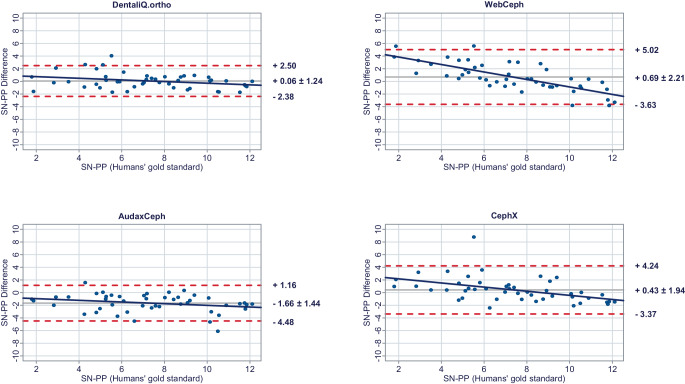
Bland–Altman plots comparing the analyses of the commercial providers to the humans’ gold standard: SN-PP Bland-Altman-Plots zum Vergleich der Analysen der kommerziellen Anbieter mit dem menschlichen Goldstandard: SN-PP

**Fig. 5:**
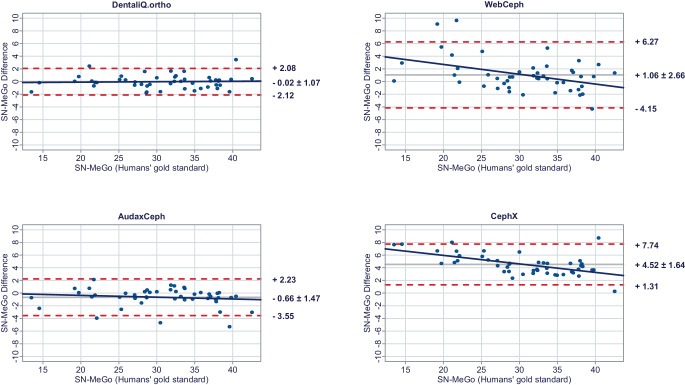
Bland–Altman plots comparing the analyses of the commercial providers to the humans’ gold standard: SN-MeGo Bland-Altman-Plots zum Vergleich der Analysen der kommerziellen Anbieter mit dem menschlichen Goldstandard: SN-MeGo

**Fig. 6:**
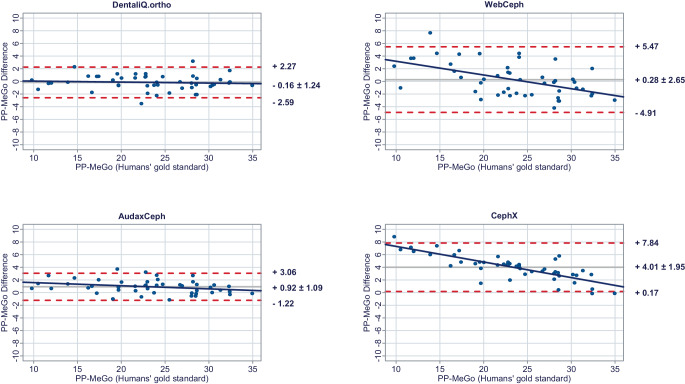
Bland–Altman plots comparing the analyses of the commercial providers to the humans’ gold standard: PP-MeGo Bland-Altman-Plots zum Vergleich der Analysen der kommerziellen Anbieter mit dem menschlichen Goldstandard: PP-MeGo

**Fig. 7:**
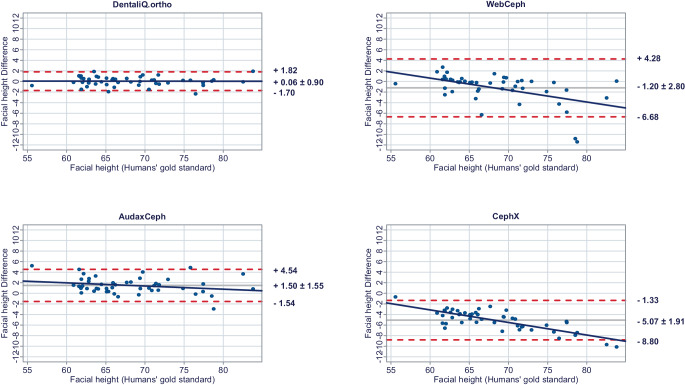
Bland–Altman plots comparing the analyses of the commercial providers to the humans’ gold standard: Facial height Bland-Altman-Plots zum Vergleich der Analysen der kommerziellen Anbieter mit dem menschlichen Goldstandard: Gesichtshöhenverhältnis

**Fig. 8:**
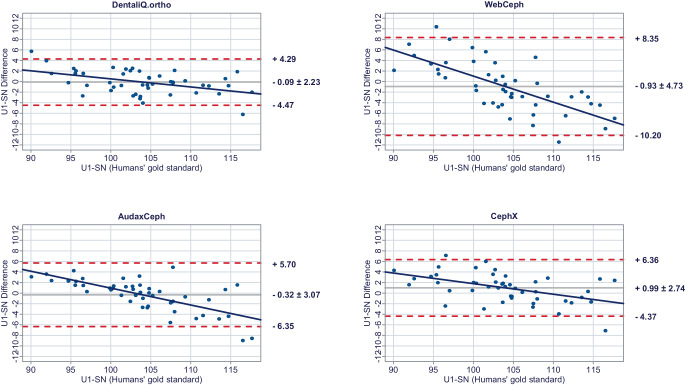
Bland–Altman plots comparing the analyses of the commercial providers to the humans’ gold standard: U1-SN Bland-Altman-Plots zum Vergleich der Analysen der kommerziellen Anbieter mit dem menschlichen Goldstandard: U1-SN

**Fig. 9:**
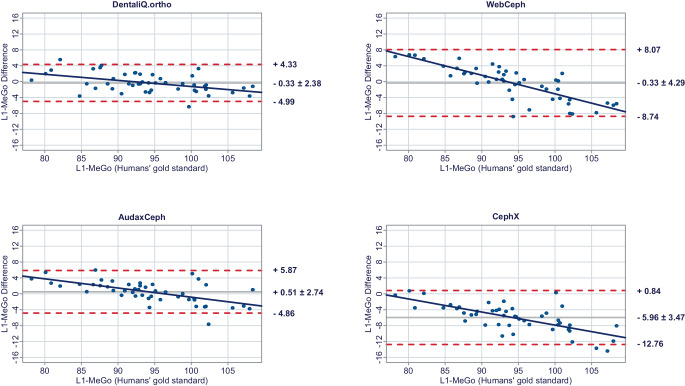
Bland–Altman plots comparing the analyses of the commercial providers to the humans’ gold standard: L1-MeGo Bland-Altman-Plots zum Vergleich der Analysen der kommerziellen Anbieter mit dem menschlichen Goldstandard: L1-MeGo

#### Skeletal sagittal parameters

DentaliQ.ortho achieved good average trueness for all skeletal sagittal parameters. Also, in terms of precision, the Bland–Altman plots demonstrate good results for DentaliQ.ortho’s AI. The risk of proportional bias can be considered to be low.

WebCeph’s AI also achieved good average trueness of its predictions for all skeletal sagittal parameters. Compared to the other providers, the LoA encompass a significantly enlarged area, so that lower precision of the results can be assumed. However, good precision can be assumed for the ANB-angle and moderate precision for SNA- and SNB-angle. Furthermore, the Bland–Altman plots show that the analyses of WebCeph have an increased risk of proportional bias.

The average trueness of AudaxCeph’s predictions was significantly lower compared to the other providers for the SNA- and SNB-angle. The mean difference compared to the humans’ gold standard was +1.36 ± 1.29° and +1.10 ± 1.14°, respectively. These analyses therefore demonstrated only low average trueness. For the ANB-angle, good average trueness was achieved and the LoA proved good precision of the predictions as well.

CephX’s AI demonstrated good average trueness for all skeletal sagittal parameters. Moreover, the LoA demonstrate good results in terms of precision for all skeletal sagittal parameters. There was a low risk of proportional bias.

#### Skeletal vertical parameters

For the skeletal vertical parameters, DentaliQ.ortho achieved good average trueness of their predictions with an average deviation from the humans’ gold standard of equal or less than ∆0.16° for all four parameters analyzed. The limits of agreement cover a narrow range, so that good precision was also demonstrated. Moreover, the Bland–Altman plots show that there was almost no risk of proportional bias.

WebCeph’s AI only demonstrated good average trueness for PP-MeGo. For the other three skeletal vertical parameters, the average deviation of the AI predictions and the humans’ gold standard was +0.69 ± 2.21° for SN-PP, +1.06 ± 2.66° for SN-MeGo, and −1.20 ± 2.80% for facial height so that only moderate average trueness was achieved for SN-PP and low average trueness for SN-MeGo and facial height, respectively. Moreover, precision of WebCeph’s AI was lowest compared to the other providers. There was an increased risk of proportional bias for all four investigated parameters.

Only moderate average trueness of AudaxCephs’ predictions was found for the parameters SN-MeGo and PP-MeGo and low average trueness for the parameters SN-PP and facial height. The average deviation of the AI predictions from to the humans’ gold standard ranged from −0.66 ± 1.47° (SN-MeGo) to −1.66 ± 1.44° (SN-PP). However, the precision of the predictions was good for SN-PP, SN-MeGo, and PP-MeGo, respectively, and moderate for facial height. The risk of proportional bias was low for all four parameters.

CephX’s AI only demonstrated good average trueness for the parameter SN-PP. For the other three skeletal vertical parameters, a very large deviation between the predictions of the AI and the humans’ gold standard of +4.52 ± 1.64° for SN-MeGo, +4.01 ± 1.95° for PP-MeGo, and −5.07 ± 1.91% for the facial height was found, so that very poor average trueness must be assumed. Moreover, the risk of proportional bias for all parameters was increased.

#### Dental parameters

DentaliQ.ortho’s AI was able to achieve good average trueness for both the inclination of the upper and the lower incisors. Precision can be considered moderate with the LoAs ranging from −4.47° to +4.29° for U1-SN and −4.99° to +4.33° for L1-MeGo. The risk of proportional bias was moderate as well.

WebCephs’ AI exhibited moderate average trueness for the inclination of the upper incisors and good average trueness for the lower incisors. However, with LoAs ranging from −10.20 to +8.35° for the inclination of the upper incisors and −8.74 to +8.07° for the inclination of the lower incisors, the precision of the AI must be considered very low. Moreover, the risk of proportional bias was very high for both parameters.

Good average trueness was demonstrated by AudaxCeph’s AI for the inclination of the upper incisors and moderate average trueness for the inclination of the lower incisors. However, LoAs between −6.35 and 5.70° for the inclination of the upper incisors and −4.86 and +5.87° for the inclination of the lower incisors demonstrated only low precision. The risk of proportional bias was moderate.

While CephX’s AI achieved moderate average trueness for the inclination of the upper incisors, average trueness of the predictions for the lower incisors was very low with an average deviation of −5.96 ± 3.47° compared to the humans’ gold standard. The precision for both parameters can be regarded as low and the risk of proportional bias as moderate.

## Discussion

In recent years, many efforts have been made to integrate AI into orthodontic diagnosis and treatment planning. The majority of currently available studies in this context aim to automatize cephalometric X‑ray analysis, which is still an essential part of orthodontic diagnostics [3]. Prior to AI-assisted analyses, locating the landmarks on cephalometric X‑rays as a basis for geometric constructions and measurements used to be a time-consuming and often error-prone process. Recently, several research groups have been able to automatize this manual process using different AI algorithms [1, 5, 7, 9, 12–14, 17–20, 25, 27, 28, 32, 33, 37].

The majority of studies published to date that investigated the use of AI for automated analysis of cephalometric X‑ray images assessed the accuracy of AI based on the metric deviation between landmarks set by the AI and a human gold standard. The literature claims a difference of 2 mm to be clinically sufficiently precise and therefore acceptable [1, 6, 9, 20, 24, 25, 27, 28, 32, 36]. In a meta-analysis published in 2021, Schwendicke et al. evaluated the accuracy of automated cephalometric landmark detection by various authors and were able to show that the majority of the included studies was capable to locate the landmarks within this 2 mm tolerance limit [31]. However, the authors pointed out that there is an increased risk of bias in a large number of the studies included in this meta-analysis.

Meanwhile, the enormous potential of automated cephalometric analysis has been recognized not only by clinicians in terms of saving time and quality management, but also by various companies with the aim of commercializing such service. The underlying data used for creation of the models on which these services rely on is often unclear and not presented in any detail [31]. Therefore, the aim of this investigation was to evaluate the accuracy and quality of all providers for automated cephalometric analysis that met the defined inclusion criteria at the time of data collection and to compare their quality to a high-level gold standard.

At present, the analysis of cephalometric images by human experts can be considered as gold standard [17]. Nevertheless, in spite of clear definitions for the landmarks, it must be assumed that even the evaluation by human experts can be prone to errors [8, 10, 21, 34]. To achieve a high level of quality for our gold standard, a set of 50 different randomly selected cephalometric X‑rays was compiled. The median value of the evaluations of 12 different human orthodontic specialists was defined as the humans’ gold standard for each examined parameter. Hereby, outliers of the humans’ analyses were ruled out. All other existing studies evaluating commercially available automated cephalometric analyses lack a qualitatively comparable humans’ gold standard [21–23, 29, 35]. Moreover, unlike most studies, we decided not to assess the quality of the automated cephalometric analyses based on the accuracy of the detected landmarks, as the effective clinical accuracy of orthodontic parameters is not only given by the metric deviation of the landmarks, but also by the direction of such deviation [26, 30]. For example, in case of angular measurements, inaccuracies of the landmarks along the angular legs do not lead to any change in the resulting orthodontic parameters [17]. Therefore, as demanded by Santoro et al., we performed the comparisons of the different providers with the humans’ gold standard based on the resulting orthodontic parameters themselves [30].

For the evaluation of an analysis’ accuracy in comparison to a gold standard, it is necessary to provide information about both the average trueness and the precision of the analysis. Average trueness describes the level of agreement between the arithmetic mean of a large number of test results and the reference value, whereas precision refers to the degree of agreement between different test results. Only if good quality can be proven for both factors, good accuracy of a given analysis can be assumed. The Bland–Altman plots used in this study are suitable for evaluating both factors and for direct comparison of the analyses of the different providers. Furthermore, proportional biases can be visualized by such plots by adding linear regression lines.

Our results show that there are major differences in the quality of evaluation between different providers as well as between different parameters. While no significant mean differences were found for any of the parameters of DentaliQ.ortho and WebCeph, five out of nine parameters of CephX and even seven out of nine parameters of AudaxCeph showed significant deviations in comparison to the humans’ gold standard. It should be highlighted that particularly large mean differences to the humans’ gold standard of up to almost 6° were found for four parameters (SN-MeGo, PP-MeGo, facial height, L1-MeGo) in the CephX’s predictions. The most obvious explanation would be that all these values rely on the “gonion” landmark, for which multiple different definitions exist in literature. Should the provider rely on a differing method of determining “gonion”, large deviations should be expected. After consultation with the provider, however, this explanation was ruled out.

In general, a reduced precision of the AI predictions must be expected for dental parameters determining the inclination of the incisors. This fact has already been noted by several authors and could be explained by the fact that many structures radiologically superimpose the area of the landmarks required to determine the inclination of the anterior teeth. Moreover, these landmarks show an increased variability even when pinpointed by human experts [2, 4, 17, 22]. Since all AI algorithms are built on training data provided by humans, reduced precision for these parameters is the logical result. Nevertheless, the accuracy of the dental parameters, in particular those provided by DentaliQ.ortho and AudaxCeph, as well as the maxillary incisor inclination of CephX, can still be considered to be clinically acceptable. In contrast, clinically acceptable accuracy cannot be assumed for the dental analyses of WebCeph, as well as for the mandibular incisor inclination of CephX.

To date, only a very limited number of studies that deal with the evaluation of various commercial providers are available. None of them compared several different providers to each other and to a properly defined gold standard [21–23, 29, 35]. Therefore, the discussion of this study’s results in context with existing literature is limited to very few previously published investigations. The high quality of the cephalometric analyses performed by DentaliQ.ortho has been previously described [17]. In this study, this AI was able to perform cephalometric analyses at almost the same level as human experts. The results of our study confirm the high accuracy of the AI evaluations performed by DentaliQ.ortho. Moreover, Moreno and Gebeile-Chauty (2022) investigated and compared the accuracy of the landmarks pinpointed by the AI of DentaliQ.ortho and WebCeph [23]. The authors demonstrated a slight superiority of the analyses performed by the AI of DentaliQ.ortho although the differences were statistically not significant. Mahto et al. (2022) compared the accuracy of WebCeph’s AI to a single human expert and concluded that the automated cephalometric measurements obtained from WebCeph were fairly accurate [21]. Also in 2022, Yassir et al. [35] evaluated the cephalometric analyses of WebCeph in a similar way as Mahto et al. The Bland–Altman plots of their study depict similar results in comparison to our study. The authors recommend using WebCeph’s automated cephalometric analysis with caution and only accompanied by checks by a clinician. Findings by Kilinc et al., who were able to demonstrate significant differences between the analyses of WebCephs’ AI and a humans’ gold standard, support these conclusions [11]. For the AI of AudaxCeph, Ristau et al. (2022) published a study that showed significant differences to the humans’ gold standard for only two out of thirteen landmarks [29]. Although no direct comparison to the present study is possible as Ristau et al. did not evaluate the accuracy based on the orthodontic parameters, these results seem to contradict in some way the many significant differences to the humans’ gold standard in the present study. Meric and Naoumova (2020) investigated the accuracy of CephX’s AI compared to a human gold standard using twelve orthodontic parameters [22]. The authors conclude that CephX’s AI did not produce results of sufficient quality and that improvement of the AI was needed. These results are consistent with our findings, as we also found some significant deviations from our humans’ gold standard for the AI of CephX.

## Conclusion

In recent years, artificial intelligence (AI) has allowed various scientific groups to fully automatize cephalometric analysis and the first companies offering fully automated systems for this purpose have entered the market. The present study compared the accuracy of these commercial analyses to a high-level humans’ gold standard. Our results show that there are major differences between the assessment qualities of the different providers, with DentaliQ.ortho’s AI achieving the best results in terms of analysis’ accuracy. Furthermore, it was shown that accuracy is reduced for the parameter incisor’s inclination for all investigated AIs. Finally, it can be concluded that fully automated cephalometric analyses are promising in terms of timesaving and possible avoidance of individual human errors; however, they should presently only be used clinically under supervision by experienced clinicians.

## References

[1] Arik SO, Ibragimov B, Xing L (2017) Fully automated quantitative cephalometry using convolutional neural networks. J Med Imaging 4(1):14501. 10.1117/1.JMI.4.1.014501 10.1117/1.JMI.4.1.014501 PMC5220585 28097213

[2] Baumrind S, Frantz RC (1971) The reliability of head film measurements. 2. Conventional angular and linear measures. Am J Orthod 60(5):505–17. 10.1016/0002-9416(71)90116-3 5286677 10.1016/0002-9416(71)90116-3

[3] Broadbent B (1931) A new X‑ray technique and its application to orthodontia. Angle Orthod 1(2):45–66

[4] Chan CK, Tng TH, Hägg U, Cooke MS (1994) Effects of cephalometric landmark validity on incisor angulation. Am J Orthod Dentofacial Orthop 106(5):487–495. 10.1016/s0889-5406(94)70071-0 7977189 10.1016/S0889-5406(94)70071-0

[5] Chen R, Ma Y, Chen N, Lee D, Wang W (2019) Cephalometric landmark detection by attentivefeature pyramid fusion and regression-voting. MICCAI. 10.48550/arXiv.1908.08841

[6] Dai X, Zhao H, Liu T, Cao D, Xie L (2019) Locating anatomical landmarks on 2D lateral cephalograms through adversarial encoder-decoder networks. IEEE Access 7:132738–132747. 10.1109/ACCESS.2019.2940623

[7] Gilmour L, Ray N (2020) Locating cephalometric X‑Ray landmarks with foveated pyramid attention. MIDL. 10.48550/arXiv.2008.04428

[8] Gonçalves FA, Schiavon L, Pereira Neto JS, Nouer DF (2006) Comparison of cephalometric measurements from three radiological clinics. Braz Oral Res 20(2):162–166. 10.1590/S1806-83242006000200013 16878211 10.1590/s1806-83242006000200013

[9] Hwang HW, Park JH, Moon JH, Yu Y, Kim H, Her SB, Srinivasan G, Aljanabi MNA, Donatelli RE, Lee SJ (2020) Automated identification of cephalometric landmarks: part 2—Might it be better than human? Angle Orthod 90(1):69–76. 10.2319/022019-129.1 31335162 10.2319/022019-129.1 PMC8087057

[10] Kamoen A, Dermaut L, Verbeeck R (2001) The clinical significance of error measurement in the interpretation of treatment results. Eur J Orthod 23(5):569–578. 10.1093/ejo/23.5.569 11668876 10.1093/ejo/23.5.569

[11] Kılınç DD, Kırcelli BH, Sadry S, Karaman A (2022) Evaluation and comparison of smartphone application tracing, web based artificial intelligence tracing and conventional hand tracing methods. J Stomatol Oral Maxillofac Surg 123(6):e906–e915. 10.1016/j.jormas.2022.07.017 35901950 10.1016/j.jormas.2022.07.017

[12] Kim H, Shim E, Park JM, Kim Y‑J, Lee U‑T, Kim Y (2020) Web-based fully automated cephalometric analysis by deep learning. Comput Methods Programs Biomed 194:105513 32403052 10.1016/j.cmpb.2020.105513

[13] Kim J, Kim I, Kim YJ, Kim M, Cho JH, Hong M, Kang KH, Lim SH, Kim SJ, Kim YH, Kim N, Sung SJ, Baek SH (2021) Accuracy of automated identification of lateral cephalometric landmarks using cascade convolutional neural networks on lateral cephalograms from nationwide multi-centres. Orthod Craniofac Res 24(Suppl 2):59–67. 10.1111/ocr.12493 33973341 10.1111/ocr.12493

[14] Kim YH, Lee C, Ha E‑G, Choi YJ, Han S‑S (2021) A fully deep learning model for the automatic identification of cephalometric landmarks. Imaging Sci Dent 51:299–306 34621657 10.5624/isd.20210077 PMC8479429

[15] Krouwer JS (2008) Why Bland-Altman plots should use X, not (Y+X)/2 when X is a reference method. Stat Med 27(5):778–780. 10.1002/sim.3086 17907247 10.1002/sim.3086

[16] Kunz F, Stellzig-Eisenhauer A (2022) Künstliche Intelligenz in der Kieferorthopädie. Quintessenz Zahnmed 9:836–841

[17] Kunz F, Stellzig-Eisenhauer A, Zeman F, Boldt J (2020) Artificial intelligence in orthodontics: evaluation of a fully automated cephalometric analysis using a customized convolutional neural network. J Orofac Orthop 81(1):52–68. 10.1007/s00056-019-00203-8 31853586 10.1007/s00056-019-00203-8

[18] Le VNT, Kang J, Oh IS, Kim JG, Yang YM, Lee DW (2022) Effectiveness of human-artificial intelligence collaboration in cephalometric landmark detection. J Pers Med 12(3):387. 10.3390/jpm12030387 35330386 10.3390/jpm12030387 PMC8954049

[19] Lee C, Tanikawa C, Lim J‑Y, Yamashiro T (2019) Deep learning based cephalometric landmark identification using landmark-dependent multi-scale patches

[20] Lee J‑H, Yu H‑J, Kim M‑J, Kim JW, Choi J (2020) Automated cephalometric landmark detection with confidence regions using bayesian convolutional neural networks. BMC Oral Health 20(1):270. 10.1186/s12903-020-01256-7 33028287 10.1186/s12903-020-01256-7 PMC7541217

[21] Mahto RK, Kafle D, Giri A, Luintel S, Karki A (2022) Evaluation of fully automated cephalometric measurements obtained from web-based artificial intelligence driven platform. BMC Oral Health 22(1):132. 10.1186/s12903-022-02170-w 35440037 10.1186/s12903-022-02170-w PMC9020017

[22] Meriç P, Naoumova J (2020) Web-based fully automated cephalometric analysis: comparisons between app-aided, computerized, and manual tracings. Turk J Orthod 33(3):142–149. 10.5152/TurkJOrthod.2020.20062 32974059 10.5152/TurkJOrthod.2020.20062 PMC7491969

[23] Moreno M, Gebeile-Chauty S (2022) Comparative study of two software for the detection of cephalometric landmarks by artificial intelligence. Orthod Fr 93(1):41–61. 10.1684/orthodfr.2022.73 35785943 10.1684/orthodfr.2022.73

[24] Nishimoto S, Sotsuka Y, Kawai K, Ishise H, Kakibuchi M (2019) Personal computer-based cephalometric landmark detection with deep learning, using cephalograms on the Internet. J Craniofac Surg 30(1):91–95. 10.1097/SCS.0000000000004901 30439733 10.1097/SCS.0000000000004901

[25] Oh K, Oh IS, Le VNT, Lee DW (2021) Deep anatomical context feature learning for cephalometric landmark detection. IEEE J Biomed Health Inform 25(3):806–817. 10.1109/JBHI.2020.3002582 32750939 10.1109/JBHI.2020.3002582

[26] Ongkosuwito EM, Katsaros C, van’t Hof MA, Bodegom JC, Kuijpers-Jagtman AM (2002) The reproducibility of cephalometric measurements: a comparison of analogue and digital methods. Eur J Orthod 24(6):655–665. 10.1093/ejo/24.6.655 12512783 10.1093/ejo/24.6.655

[27] Park J‑H, Hwang H‑W, Moon J‑H, Yu Y, Kim H, Her S‑B, Srinivasan G, Aljanabi M, Donatelli R, Lee S‑J (2019) Automated identification of cephalometric landmarks: part 1—Comparisons between the latest deep-learning methods YOLOV3 and SSD. Angle Orthod. 10.2319/022019-127.1 31282738 10.2319/022019-127.1 PMC8109157

[28] Qian J, Luo W, Cheng M, Tao Y, Lin J, Lin H (2020) CephaNN: a multi-head attention network for cephalometric landmark detection. IEEE Access 8:112633–112641. 10.1109/ACCESS.2020.3002939

[29] Ristau B, Coreil M, Chapple A, Armbruster P, Ballard R (2022) Comparison of AudaxCeph®’s fully automated cephalometric tracing technology to a semi-automated approach by human examiners. Int Orthod 20(4):100691. 10.1016/j.ortho.2022.100691 36114136 10.1016/j.ortho.2022.100691

[30] Santoro M, Jarjoura K, Cangialosi TJ (2006) Accuracy of digital and analogue cephalometric measurements assessed with the sandwich technique. Am J Orthod Dentofacial Orthop 129(3):345–351. 10.1016/j.ajodo.2005.12.010 16527629 10.1016/j.ajodo.2005.12.010

[31] Schwendicke F, Chaurasia A, Arsiwala L, Lee JH, Elhennawy K, Jost-Brinkmann PG, Demarco F, Krois J (2021) Deep learning for cephalometric landmark detection: systematic review and meta-analysis. Clin Oral Investig 25(7):4299–4309. 10.1007/s00784-021-03990-w 34046742 10.1007/s00784-021-03990-w PMC8310492

[32] Song Y, Qiao X, Iwamoto Y, Chen Y (2020) Automatic cephalometric landmark detection on X‑ray images using a deep-learning method. Appl Sci 10(7):2547. 10.3390/app10072547

[33] Tanikawa C, Lee C, Lim J, Oka A, Yamashiro T (2021) Clinical applicability of automated cephalometric landmark identification: part I—Patient-related identification errors. Orthod Craniofac Res 24(Suppl 2):43–52. 10.1111/ocr.12501 34021976 10.1111/ocr.12501

[34] Wang CW, Huang CT, Hsieh MC, Li CH, Chang SW, Li WC, Vandaele R, Maree R, Jodogne S, Geurts P, Chen C, Zheng G, Chu C, Mirzaalian H, Hamarneh G, Vrtovec T, Ibragimov B (2015) Evaluation and comparison of anatomical landmark detection methods for cephalometric X‑Ray images: a grand challenge. IEEE Trans Med Imaging 34(9):1890–1900. 10.1109/TMI.2015.2412951 25794388 10.1109/TMI.2015.2412951

[35] Yassir YA, Salman AR, Nabbat SA (2022) The accuracy and reliability of WebCeph for cephalometric analysis. J Taibah Univ Med Sci 17(1):57–66. 10.1016/j.jtumed.2021.08.010 35140566 10.1016/j.jtumed.2021.08.010 PMC8801471

[36] Zeng M, Yan Z, Liu S, Zhou Y, Qiu L (2021) Cascaded convolutional networks for automatic cephalometric landmark detection. Med Image Anal 68:101904. 10.1016/j.media.2020.101904 33290934 10.1016/j.media.2020.101904

[37] Zhong Z, Li J, Zhang Z, Jiao Z, Gao X (2019) An attention-guided deep regression model for landmark detection in cephalograms, pp 540–548

